# DINO-Mix enhancing visual place recognition with foundational vision model and feature mixing

**DOI:** 10.1038/s41598-024-73853-3

**Published:** 2024-09-27

**Authors:** Gaoshuang Huang, Yang Zhou, Xiaofei Hu, Chenglong Zhang, Luying Zhao, Wenjian Gan

**Affiliations:** Institute of Geospatial Information, PLA Strategic Support Force Information Engineering University, Zhengzhou, 450001 China

**Keywords:** Visual place recognition, DINOv2, Feature mixer, Foundational vision model, Image retrieval, Engineering, Image processing, Learning algorithms, Network models

## Abstract

Using visual place recognition (VPR) technology to ascertain the geographical location of publicly available images is a pressing issue. Although most current VPR methods achieve favorable results under ideal conditions, their performance in complex environments, characterized by lighting variations, seasonal changes, and occlusions, is generally unsatisfactory. Therefore, obtaining efficient and robust image feature descriptors in complex environments is a pressing issue. In this study, we utilized the DINOv2 model as the backbone for trimming and fine-tuning to extract robust image features and employed a feature mix module to aggregate image features, resulting in globally robust and generalizable descriptors that enable high-precision VPR. We experimentally demonstrated that the proposed DINO-Mix outperforms the current state-of-the-art (SOTA) methods. Using test sets having lighting variations, seasonal changes, and occlusions such as Tokyo24/7, Nordland, and SF-XL-Testv1, our proposed architecture achieved Top-1 accuracy rates of 91.75%, 80.18%, and 82%, respectively, and exhibited an average accuracy improvement of 5.14%. In addition, we compared it with other SOTA methods using representative image retrieval case studies, and our architecture outperformed its competitors in terms of VPR performance. Furthermore, we visualized the attention maps of DINO-Mix and other methods to provide a more intuitive understanding of their respective strengths. These visualizations serve as compelling evidence of the superiority of the DINO-Mix framework in this domain.

## Introduction

Visual place recognition (VPR), also known as image geo-localization (IG) or visual geo-localization, has been extensively applied in several fields, such as augmented reality^1^, robotics^2^, autonomous driving^2,3^, object tracking^4^, 3d reconstruction^5^, and intelligence gathering^6^. Currently, most VPR studies focus on image retrieval in urban scenarios. However, urban environment images may include many conditions, such as multiple shooting angles, illumination changes, seasonal variations, and occlusions, that can affect the successful retrieval of high-precision image features. Therefore, extracting robust and generalizable image feature descriptors for accurate image retrieval is critical.

In previous approaches to VPR, handcrafted scale-invariant feature transform (SIFT)^7^, histogram of oriented gradient (HOG)^8^, speeded-up robust features (SURF)^9^, oriented FAST and BRIEF (ORB)^10^, and other techniques have been used to extract features from images. These features were then aggregated using methods such as bag-of-words^11^, fisher vector^12^, and vector of locally aggregated descriptors (VLAD)^13^ to obtain image descriptors for image retrieval, enabling IG. More recently, deep-learning techniques have emerged as mainstream approaches for extracting image features^14–16^. These methods significantly improve VPR accuracy compared with handcrafted features. Examples include NetVLAD^17^, combining convolutional neural networks (CNNs) with VLAD, and other variants incorporating attention, semantics, context, and multiscale features. Other methods based on the generalized mean (GeM)^18^, such as CosPlace^19^, and those using fully-connected multilayer perceptron (MLP)-based feature aggregation, such as MixVPR^20^, have been proposed. However, these methods either use only handcrafted features or use models that have not been trained with large amounts of data as the backbone and are less robust. In practical testing, the accuracy of these methods for IG has been suboptimal under challenging conditions, such as different shooting angles, illumination changes, seasonal variations, and occlusions.

The rapid development of foundational visual models has enabled the generation of universal visual features from images^21^. By training on billions of data points, foundational visual models can extract image features that are more generalizable and robust than those extracted by conventional models. They can effectively handle the challenging conditions encountered in practice. Therefore, incorporating foundational visual models into VPR is a promising approach.

Considering the abovementioned challenges, this study proposes a novel VPR framework based on the DINOv2^21^ model, called DINO-Mix, which combines foundational visual models with feature aggregation. This framework possesses exceptional discriminative power. Efficient and robust image features suitable for IG are extracted by fine-tuning and trimming. Furthermore, it uses a feature mixer^22^ module to aggregate image features, resulting in a global feature descriptor vector. DINO-Mix is experimentally demonstrated to achieve superior test accuracy on multiple benchmarks, surpassing state-of-the-art (SOTA) methods. The contributions of our work are as follows:

1. We propose a novel VPR framework, DINO-Mix. The framework combines the excellent feature extraction capability of the foundational vision model and the feature aggregation capability of the feature mix module, which allows the framework to easily cope with challenges such as viewpoint changes, illumination changes, seasonal changes, and occlusion.
2. We conduct a series of ablation experiments to pinpoint the optimal parameters for our framework, including the optimal number of mix layers, descriptor dimension, and backbone architecture. The experimental results demonstrate that the test accuracies of our framework with optimal parameters exceed those of existing SOTA methods.
3. We present illustrative examples of VPR for our framework and other approaches and perform heat map visualizations to highlight the advantages of our framework.

The remainder of this paper is organized as follows. In Sect. 2, we summarize previous relevant research on visual place recognition. In Sect. 3, the DINO-Mix framework is introduced, and we provide details of the training set, testing set, and the training and evaluation parameters used in our experiments. Section 4 presents the results by comparing the proposed framework with existing methods in terms of accuracy and ablation experiments are conducted. We qualitatively demonstrate the DINO-Mix architecture by comparing it with other state-of-the-art methods through a typical VPR example and visualizing the corresponding attention map. Finally, our conclusions are presented in Sect. 5.

## Related work

By retrieving the most similar image from the image database, the geographical location of the retrieved image can be used as the location of the target image^23^. In recent years, numerous researchers have made significant contributions to the field of image retrieval for VPR. The features used in image retrieval can be broadly categorized into handcrafted and deep features. Zhang and Kosecka^24^ first extracted SIFT features from images to establish an image feature database. They performed a brute-force global database search and validated and ranked the top five candidate images using the random sample consensus algorithm^25^. Finally, the geographical location of the target image was obtained by triangulating the top three images. Zamir and Shah^26^ extracted SIFT feature vectors from images to build a database and used a nearest-neighbor tree search to improve retrieval efficiency. Zamir et al.^27^ further improved the nearest-neighbor matching technique by pruning outliers and applying the generalized minimum clique problem in conjunction with approximate feature matching. This resulted in a 5% improvement in localization accuracy compared to their previous study^26^. The advantages of using handcrafted features for VPR are their simplicity and strong interpretability. However, these methods tend to have high redundancy, require dimensionality reduction, are susceptible to environmental changes, and generally have low accuracy.

Deep features are extracted by neural networks with modules such as convolutional layers and attention mechanisms. These features often outperform handcrafted features owing to their strong expressive power, ability to freely define feature dimensions, and flexibility in designing neural network frameworks. Noh et al.^28^ proposed a deep local feature descriptor and an attention mechanism to identify semantic local features for key-point selection. Ng et al.^29^ introduced a global descriptor called second-order loss and attention for image retrieval that used spatial attention and descriptor similarity to perform large-scale image retrieval using second-order information. Chu et al.^30^ constructed a CNN to extract dense features, embedded an attention module within the network to score features, and proposed a grid feature point selection method to reduce the number of image features. Chu et al.^31^ combined deep with handcrafted features, extracted average pooling features from the intermediate layers of a CNN for retrieval on street-view datasets, and used SIFT to re-rank them. Yan et al.^32^ extracted hierarchical feature maps from CNNs and organically fused them for image feature representation. However, most of these methods are only based on the optimization improvement of CNN or attention modules and do not use feature aggregation modules, making it difficult to obtain robust global features.

To address environmental factors, Chu et al.^33^ used a CNN with a HOW module^34^ to extract local image features, aggregated them into a feature vector using VLAD, used the aggregated selective match kernel, and estimated the geographical location of the query image using kernel density prediction. Mishkin et al.^35^ used a bag-of-words method with multiple detectors, descriptors, and adaptive thresholds. Arandjelovic et al.^17^ designed a trainable NetVLAD layer inspired by VLAD, which provided a pooling mechanism that was integrated into other CNN structures. In addition, variants of NetVLAD have been proposed, such as CRN^36^, SPE-VLAD^37^, MultiRes-NetVLAD^38^, SARE^39^, and SFRS^40^. Ali-bey et al. proposed ConvAP^41^, which combines 1 × 1 convolutions with adaptive mean pooling to encode local features. The above methods improve the robustness of VPR in complex environments, but such methods tend to have higher computational complexity and feature vector dimensions and are more sensitive to noise, which limits the performance and accuracy of VPR.

## Methodology

### Proposed framework

**Fig. 1:**
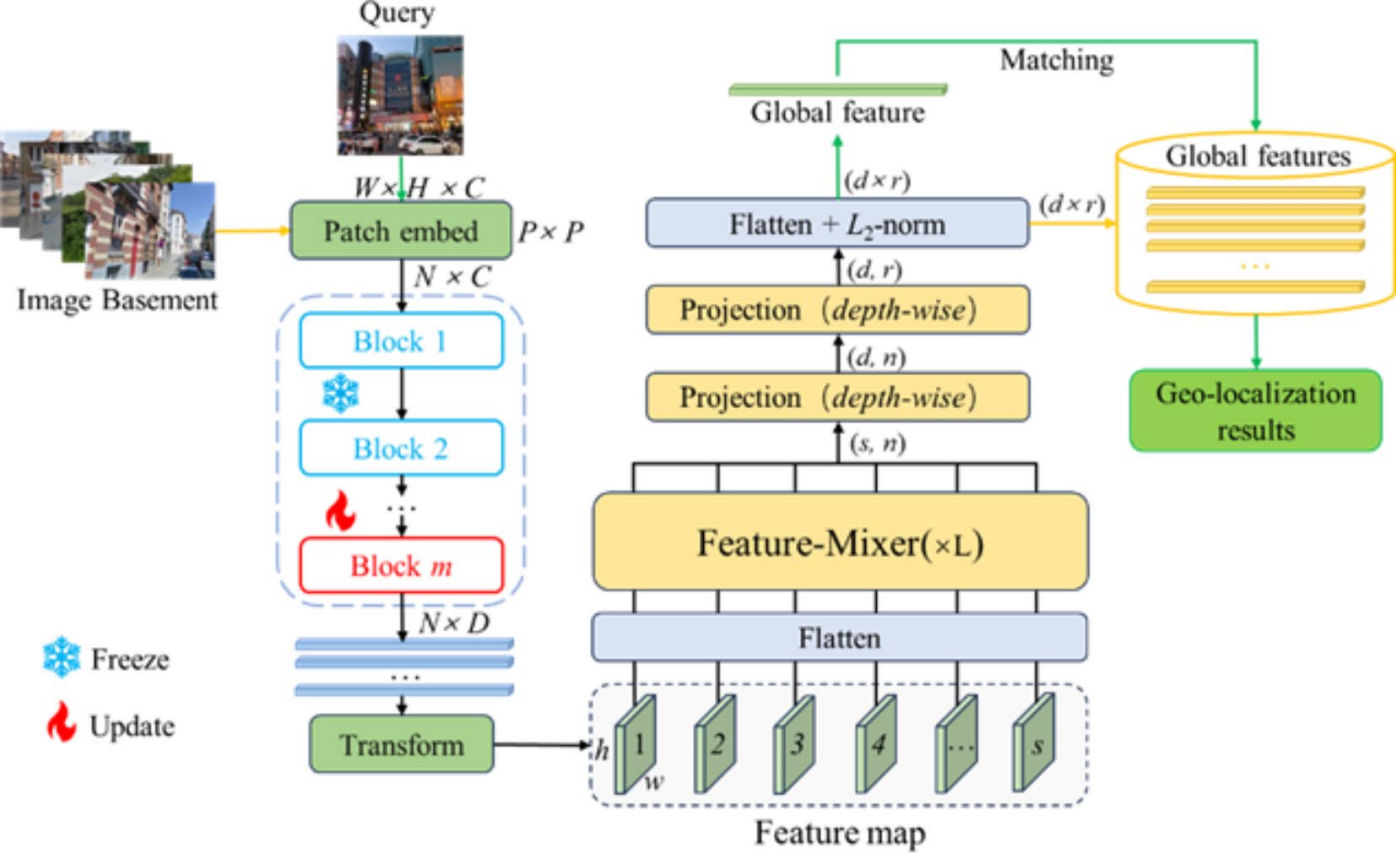
Visual place recognition (VPR) framework of DINO-Mix. The framework consists of two parts: firstly, the layer norms and head of the foundational vision model DINOv2 are pruned away and used as a backbone to extract the image feature vectors, and then the obtained feature vectors with dimensions *N × D* are transformed into *h × w × s* feature maps, and then robust global feature vectors are obtained by feature aggregation in the Mix module. During training, the front block in the backbone is frozen, and the parameters of the following few blocks and the Mix module are updated.

We aim to build robust VPR models to solve the problem of poor VPR accuracy of existing methods in scenarios such as multiple shooting angles, illumination changes, seasonal variations, and occlusions to improve the VPR accuracy. We propose a VPR framework integrating the foundational vision model with feature aggregation. The proposed framework uses the truncated DINOv2^21^ model as the backbone. DINOv2 is well-suited for several downstream tasks due to its exceptional image understanding capability. Therefore, we pre-trained the DINOv2 model as the primary network for image feature extraction and used an efficient and lightweight mixer module to aggregate the obtained image features. The DINO-Mix visual geolocation architecture is illustrated in Fig. 1.

**Fig. 2:**
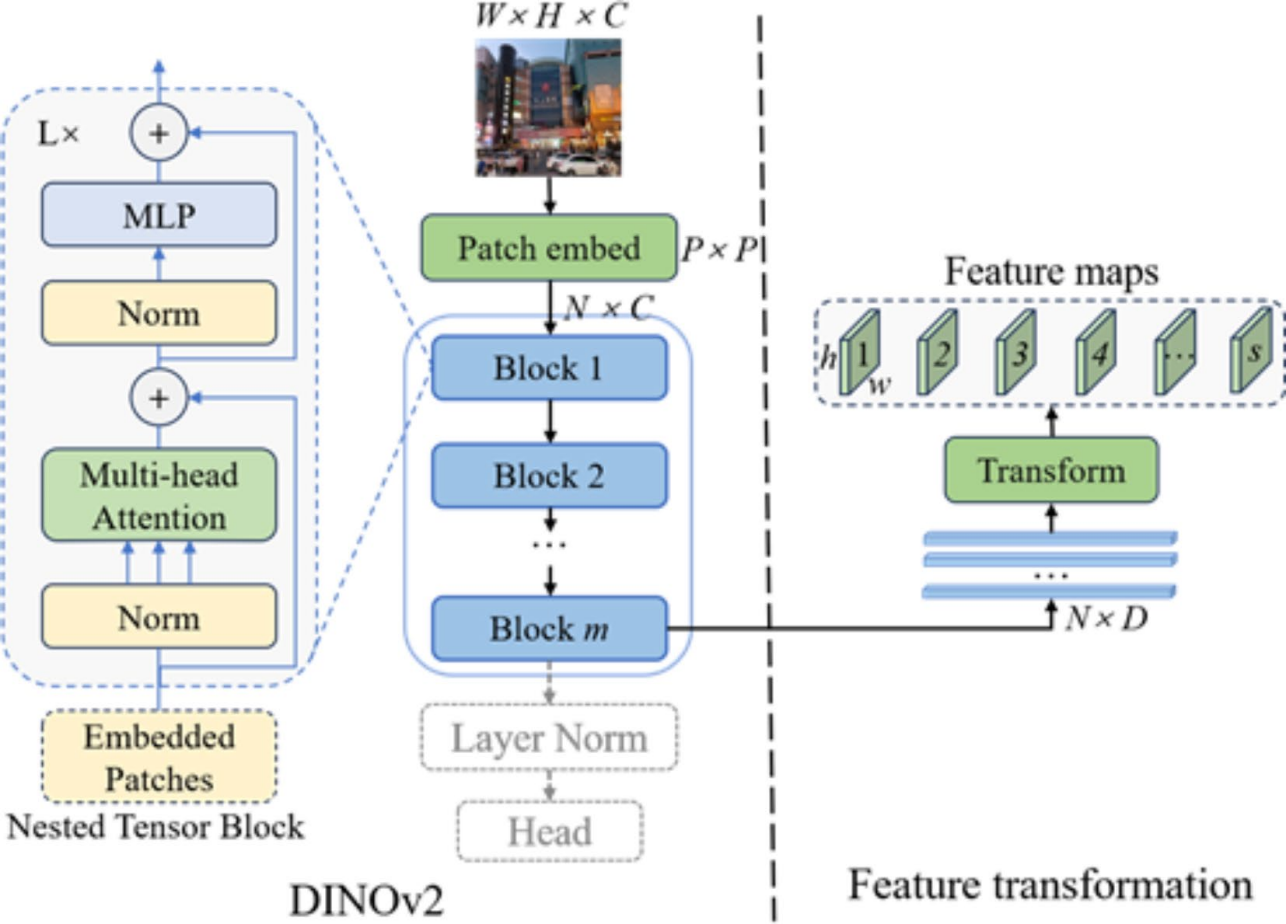
Structural diagram of the DINOv2 model and feature transformation.

We modified the DINOv2 model by removing its layer norms and head, which were subsequently used as the backbone. Furthermore, to maximize the benefits of the DINOv2 pretraining, we used the output from the last layer of the vision transformer (ViT) blocks^42^ as the input to the mixer module. Given that the output of the modified ViT block module is a feature matrix of size *N × D* (number of channels × feature vector length), we transformed it into *s* feature maps of size *h × w*, as expressed by Eq. (1). These transformed feature maps served as inputs for the mix module (see Figs. 1 and 2).

1 $$\:\left\{\begin{array}{c}\begin{array}{c}D=s\\\:N=hw\end{array}\end{array}\right.$$

where *D* is the length of the feature vector output by the backbone, *N* is the number of channels in the output of the backbone, *h* and *w* are the height and width of the feature map, respectively, and *s* is the number of feature maps.

From Fig. 1, it is evident that our DINO-Mix possesses a generalized image retrieval mode. This implies that DINO-Mix can be utilized for more than simple VPR tasks. It can identify the geographic location of a single image, enabling temporal modeling and tracking of sequential images. Moreover, it can extract ground image features to match with satellite images, facilitating cross-view image geo-localization. DINO-Mix can be employed in other domains for medical image retrieval to aid disease diagnosis and case studies. Furthermore, it can enhance the user shopping experience by applying to merchandise image retrieval and product recommendation.

### Foundational vision model: DINOv2

Foundational vision models are typically built using structures such as CNNs or transformers. These models often have tens to hundreds of millions of parameters, giving them a greater representational capacity than smaller models. In addition, because of the use of larger and more diverse datasets during training, foundational vision models can learn more features and have better generalization capabilities.

DINOv2^21^ can extract powerful image features and perform well across different tasks. DINOv2 has a broader scope of application and use areas compared with Segment Anything^43^. The architecture of the DINOv2 model is illustrated in Fig. 2. First, an input image *W × H × C* is passed through a patch embedding module consisting of a 2D convolutional layer with a kernel size of 14 × 14 and a stride of 14, followed by a normalization layer. This process uniformly outputs patches of size (*P × P*), and (*W // 14*) × (*H // 14*) is the number of channels *N*. These patches are then fed into ViT blocks, which vary in number according to the model’s size. The ViT blocks output a feature matrix of size *N* (number of channels) × *D* (dimension of the feature vector), which is then normalized by a layer-norm module before being transformed into a feature vector of size 1 × n. Finally, the head module can be flexibly selected based on specific image task requirements.

DINOv2 has several key features: (a) it presents a novel approach for training high-performance computer vision models; (b) it offers superior performance without the need for fine-tuning; (c) it can learn from any image dataset and capture certain features that existing methods struggle with; and (d) it leverages knowledge distillation to transfer knowledge from more complex teacher models to smaller student models. Through knowledge distillation, three smaller models were obtained from the ViTg14 model: ViTl14 (large), ViTb14 (base), and ViTs14 (small) (see Table 1).

**Table 1:** Four ViT model parameters for DINOv2.

Name	Patch embed	ViT blocks	Features dim	Size (MB)
ViTs14	14 × 14	12	384	86.2
ViTb14	14 × 14	12	768	338.2
ViTl14	14 × 14	24	1,024	1,189.0
ViTg14	14 × 14	40	1,536	4,439.5

The primary advantage of DINOv2 lies in its training on a large-scale dataset. This dataset, LVD-142 M, comprises 142 million images and includes ImageNet-22k, ImageNet-1k, Google Landmarks, several fine-grained datasets, and image datasets crawled from the Internet. An Nvidia A100 40-GB GPU was used for model training, with a total of 22k GPU hours dedicated to training the DINOv2-g model.

### Feature mixer

**Fig. 3:**
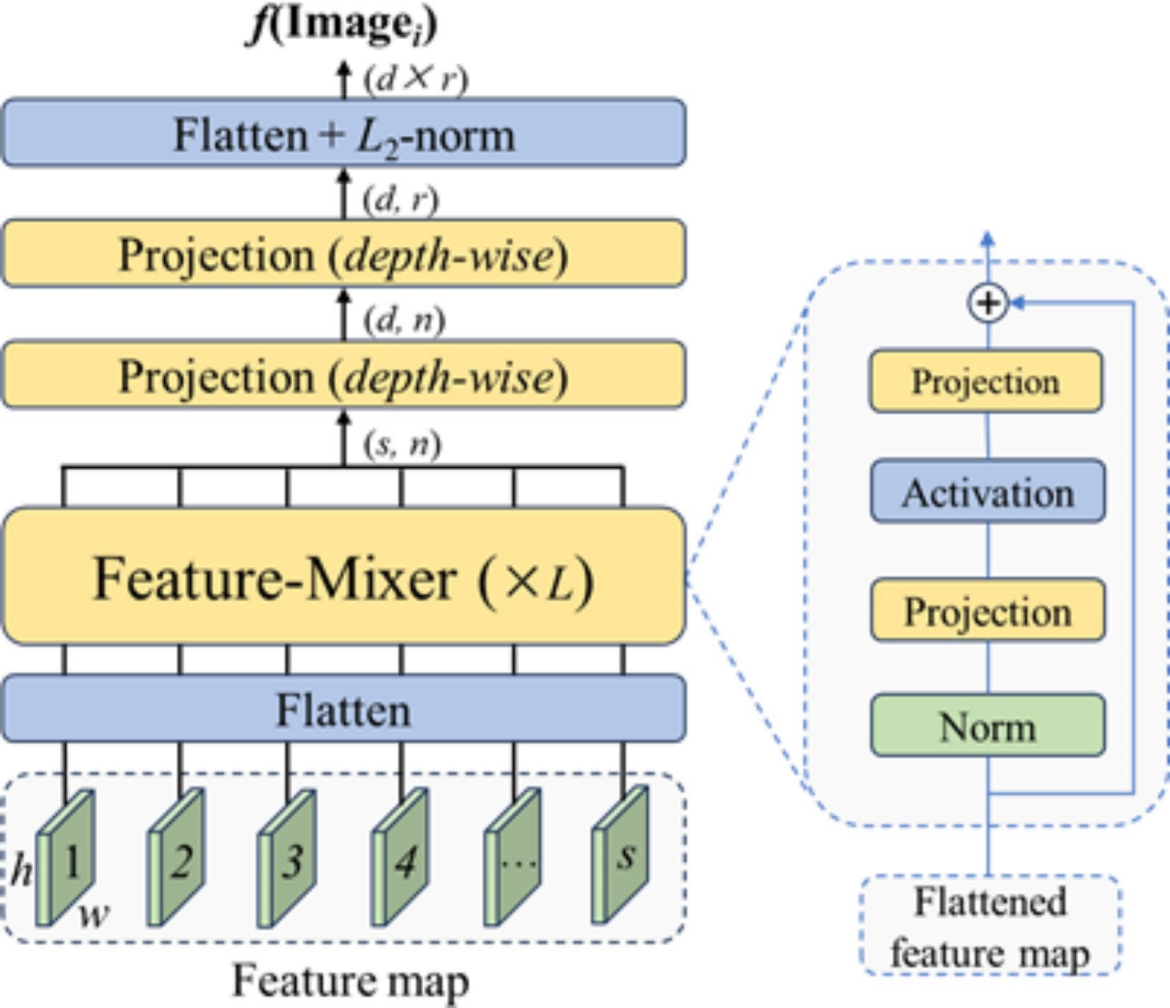
Architecture of the mixer.

Currently, the most advanced techniques propose shallow aggregation layers that are inserted into very deep pre-trained backbones cropped to the last feature-rich layer. By contrast, Wang et al. proposed TransVPR^44^, which achieved good results in local feature matching. However, its global representation performance did not surpass NetVLAD^17^ or CosPlace^19^. Recent advances in isotropic architectures have demonstrated that self-attention is not crucial for ViT. However, the mixer uses feature maps extracted from a pre-trained backbone and iteratively merges global relationships into each feature map. This is achieved through an isotropic block stack composed of MLPs, referred to as a feature mixer^22^. The effectiveness of the mixer has been demonstrated through several qualitative and quantitative results, demonstrating its high performance and lightweight nature^20^; the architecture is illustrated in Fig. 3.

The mixer treats the input feature map $\:F\in\:{R}^{s\times\:h\times\:w}$ as a set of *s* 2D features, each of size $\:h\times\:w$, as expressed by Eq. (2):

2 $$\text{F}=\left\{{X^i}\right\},\quad i=\left\{1,\dots\:,s\right\}$$

where $\:{\text{X}}^{\text{i}}$ is the *i-*th activation map in the feature map $\:F$. Secondly, each 2D feature map $\:{X}^{i}$ is expanded into a 1D vector representation, resulting in a flattened feature map $\:F\in\:{R}^{s\times\:n}$, where $\:n=h\times\:w$.

The flattened feature maps are then fed into the feature mixer, which is composed of *L* MLPs with the same structure, as shown in Fig. 3. The feature mixer takes the flattened feature map ensemble as input and successively incorporates spatial global relationships into each $\:{X}^{i}\in\:F$ as per Eq. (3):

3 $$\:{X}^{i}\leftarrow\:{W}_{2}\left(\sigma\:\left({W}_{1}{X}^{i}\right)\right)+{X}^{i},i=\left\{1,\dots\:,s\right\}$$

where $\:{W}_{1}$ and $\:{W}_{2}$ are the weights of the two fully connected layers that make up the MLP, and σ is the ReLU nonlinear activation function.

For $\:F\in\:{R}^{s\times\:n}$, the feature mixer produces an output $\:\text{Z}\in\:{R}^{s\times\:n}$ with the same shape owing to its isotropic architecture, and feeds it into the second feature mixer block and so on until *L* consecutive blocks have been traversed, as per Eq. (4):

4 $$\:Z=F{M}_{L}\left(F{M}_{L-1}\left(\dots\:F{M}_{1}\left(F\right)\right)\right)$$

where *Z* and the feature map *F* have the same dimensions, two fully connected layers are used to successfully transform the channel and row dimensions to control the dimensions of the final global descriptor. First, a depth projection is used to map Z from $\:{R}^{s\times\:n}$ to $\:{R}^{d\times\:n}$, as given by Eq. (5):

5 $$\:{Z}^{{\prime\:}}={W}_{d}\left(Transpose\left(Z\right)\right)$$

where $\:{W}_{d}$ is the weight of the fully connected layer. A row-wise projection is then used to map the output $\:{Z}^{{\prime\:}}$ from $\:{R}^{d\times\:n}$ to $\:{R}^{d\times\:r}$, as given by Eq. (6):

6 $$\:\begin{array}{c}O={W}_{r}\left(Transpose\left({Z}^{{\prime\:}}\right)\right)\end{array}$$

where $\:{W}_{r}$ is the weight of the fully connected layer. The final output *O* has dimensions of $\:d\times\:r$, which are flattened, and *L*_2_ is normalized to form a global feature vector.

### Implementation details

#### Datasets

Our model was trained using the GSV-Cities dataset^41^. The datasets used for evaluation purposes were Pittsburgh250k^45^ (contains 8k queries and 83k reference images collected from Google Street View and Pittsburgh30k-test), Pittsburgh30k-test^45^ (a subset of Pittsburgh250k, with 8k queries and 8k reference images), SF-XL-Val^19^, Tokyo24/7^46^, Nordland^47^, and SF-XL-Testv1^19^. The datasets contained extreme variations in lighting, weather, and seasons. Specific information regarding these datasets is presented in Table 2.

**Table 2:** Parameters of the training dataset and test datasets.

Dataset	Train/Val queries	Test /Test queries	Size (GB)	Database		Urban	Season	Day/ Night
				Type	Size			
GSV-Cities	524,701/0	0/0	21.7	panorama	480 × 640 300 × 400	✓	✓	✓
Pittsburgh250k	170,112/15,432	83,952/8,280	9.5	panorama	480 × 640	✓	✗	✗
Pittsburgh30k	20,000/15,024	10,000/6,816	2.0	panorama	480 × 640	✓	✗	✗
Tokyo24/7	0/0	75,984/315	4.2	panorama	480 × 640	✓	✗	✓
Nordland	0/0	27,592/27,592	1.3	front-view	360 × 640	✗	✓	✗
SF-XL-Val	0/0	8,015/7,993	0.7	panorama	512 × 512	✓	✗	✗
SF-XL-Testv1	0/0	27,191/1,000	1.3	panorama	512 × 512	✓	✗	✓

#### Architecture

We completed our experiments using two RTX 4090 GPUs, with the system powered by an Intel Xeon Platinum 8357 C CPU featuring 64 cores and equipped with SSD storage for efficient large-scale data computation. We used Pytorch as the deep-learning framework. To enable a fair comparison with other methods in terms of accuracy, we conducted precision tests on the VPR frameworks NetVLAD, GeM, ConvAP, CosPlace, and MixVPR and obtained the testing accuracy for other methods from their corresponding papers.

#### Training

Most training weights were frozen during the training process owing to the excellent pre-trained weights of the DINO-Mix backbone. However, to make it more suitable for the VPR task, we fine-tuned the end of the backbone and trained the feature aggregation module. We trained DINO-Mix following the standard framework proposed in GSV-Cities^41^, which introduced a high-precision dataset of 67k locations described by 560k images. The batch size *B* was flexibly adjusted based on the model parameter size, and each location was trained with four images, resulting in a mini-batch of *B* × 4 images. A stochastic gradient descent method^48^ with a momentum of 0.9 and a weight decay of 0.001 was used for optimization. The initial learning rate was set to 0.05 and was divided by three every five epochs. Finally, the model was trained using images resized to 224 × 224 pixels over 50 epochs. Most existing VPR studies use a triplet loss function based on weak supervision^49^ for network training; however, this approach usually has a high computational overhead. Therefore, we used multi-similarity loss^50^ as the training loss function. Multi-similarity loss mitigates large interclass and small intraclass distances in metric learning by considering multiple similarities. Instead of relying solely on absolute spatial distance as the sole metric, it uses the overall distance distribution of other sample pairs within a batch size to weigh the loss. This computational approach effectively promotes model convergence in the early stages, as expressed by Eq. (7)

7 $$\:{\mathcal{L}}_{MS}=\frac{1}{m}{\sum\:}_{i=1}^{m}\left\{\frac{1}{\alpha\:}{log}\left[1+\sum\:_{k\in\:{P}_{i}}{e}^{-\alpha\:\left({S}_{ij}-\lambda\:\right)}\right]+\frac{1}{\beta\:}{log}\left[1+\sum\:_{k\in\:{N}_{i}}{e}^{\beta\:\left({S}_{ik}-\lambda\:\right)}\right]\right\}$$

where $\:{P}_{i}$ is the set of positive sample pairs for each instance in a batch size, $\:{N}_{i}$ is the set of negative sample pairs for each instance in the same batch size, $\:{S}_{ij}$ and $\:{S}_{ik}$ are the similarities between the two images, and $\:\alpha\:$, $\:\beta\:$, and $\:\lambda\:$ are hyperparameters.

#### Evaluation

In this study, we used top-k accuracy^51^ as a metric to evaluate the precision of the VPR methods. Top-k accuracy is a commonly used evaluation method in the VPR domain, where it is considered successful if at least one of the top-k localization results for a query image has a geographical distance of less than a threshold from the true location. In our experiments, we set to 25 m to align with existing methods.

## Results and discussion

### Comparison to the SOTA

**Fig. 4:**
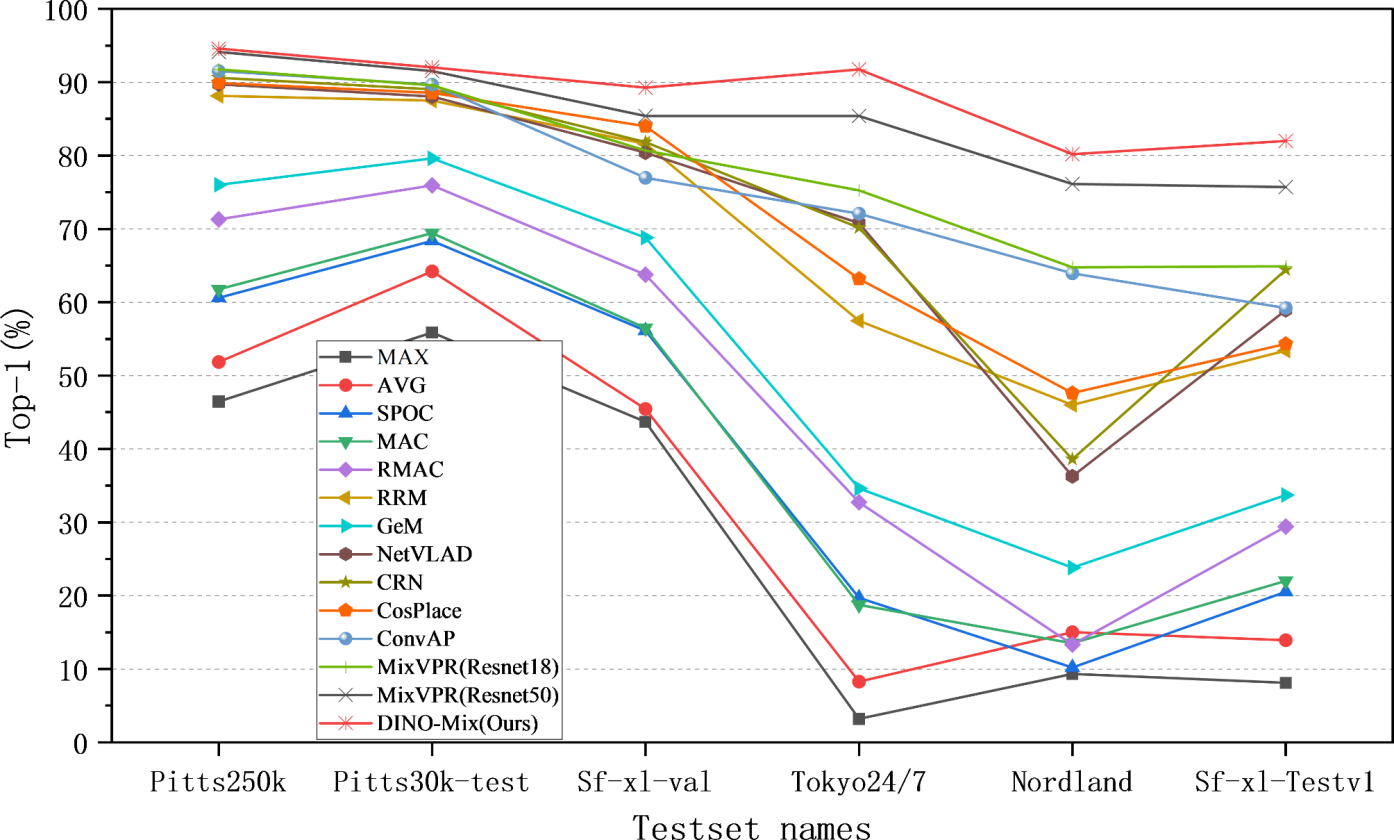
Top-1 accuracy of DINO-Mix and other VPR methods for different test sets.

We adopted the ViTb14 pre-trained model, which exhibited the best performance among the four models of DINOv2, as the backbone for DINO-Mix in the VPR task and modified the DINOv2 model by removing its layer norm and head modules. We used the mixer as a feature aggregation module to construct the model. During training, we updated the parameters of the last three blocks of the backbone and the entire mix feature aggregation module. The number of feature mixer blocks in the mix feature aggregation module was set to two, and the dimensionality of the image features output by the model was 4096. Using these optimal parameter settings, we conducted tests on six test sets for DINO-Mix and compared them with the existing methods, as shown in Table 3. In addition, Fig. 4 illustrates the difference in accuracy between DINO-Mix and other primary VPR methods.

**Table 3:** Test results of different methods on datasets with changes in viewpoint, illumination, and season. We default to using GSV-Cities as the training set, and * indicates that the method uses Pittsburgh30k as the training set.

Method	Vector dim	Test dataset (%)^a^											
		Pitts 250k		Pitts 30k		SF-XL-Val		Tokyo 24/7		Nordland		SF-XL-Testv1	
		Top1	Top5	Top1	Top5	Top1	Top5	Top1	Top5	Top1	Top5	Top1	Top5
MAX^17^	1,024	46.45	51.82	55.87	63.41	43.68	51.58	3.17	5.08	9.30	17.26	8.10	16.20
AVG^17^	1,024	51.85	58.78	64.20	73.11	45.43	53.71	8.25	8.89	14.99	26.37	13.90	22.60
SPOC^52^	256	60.59	75.77	68.37	83.45	56.11	68.75	19.68	33.33	10.18	19.17	20.50	33.50
MAC^53^	256	61.75	74.60	69.42	81.66	56.47	68.12	18.73	28.89	13.49	24.73	22.00	35.00
RMAC^54^	256	71.30	84.66	75.94	88.39	63.74	76.87	32.70	49.21	13.30	24.01	29.40	47.50
RRM^55^	256	88.14	95.12	87.49	94.15	81.60	89.67	57.46	73.33	46.00	62.77	53.40	67.40
GeM^18^	256	76.01	88.57	79.61	90.01	68.79	81.18	34.60	51.43	23.80	40.51	33.70	49.10
NetVLAD*^17^	16,384	86.93	94.07	86.36	93.27	65.34	75.60	53.97	67.62	7.86	12.55	42.50	57.90
NetVLAD^17^	16,384	89.71	96.03	88.04	94.89	80.38	88.56	70.79	80.63	36.25	51.73	58.90	72.20
CRN^36^	16,384	90.60	96.55	89.03	95.25	81.83	89.24	70.16	81.59	38.58	54.06	64.40	74.60
MultiRes-NetVLAD*^38^	32,768	86.70	93.60	86.80	93.80	—	—	69.80	81.30	—	—	—	—
SARE*^39^	4,096	88.00	94.80	87.20	93.90	—	—	74.80	84.30	—	—	45.50	56.50
SFRS*^40^	4,096	90.40	96.30	89.10	94.60	—	—	80.30	88.60	16.00	24.10	50.30	60.00
CosPlace^19^	4,096	89.89	96.14	88.54	94.75	84.01	91.33	63.17	75.87	47.62	65.11	54.30	70.60
ConvAP^41^	4,096	91.52	96.88	89.67	95.01	76.95	83.71	72.06	81.59	63.93	78.35	59.20	71.50
MixVPR (Resnet18)^20^	4,096	91.75	97.09	89.57	94.84	80.68	88.19	75.24	87.62	64.75	79.88	64.90	75.10
MixVPR (Resnet50)^20^	4,096	*94.13*	*98.18*	*91.52*	*95.47*	*87.50*	*93.43*	*85.40*	*91.43*	*76.12*	**86.81**	*75.70*	*83.1*
DINO-Mix (ViTb14) (Ours)	4,096	**94.58**	**98.86**	**92.03**	**95.89**	**89.25**	**93.50**	**91.75**	**93.97**	**80.18**	*85.44*	**82.00**	**84.90**

^a^ Numbers in bold are optimal, and those Italics are the second best for each dataset.

As listed in Table 3, the test accuracy of the DINO-Mix model proposed in this study has comprehensively surpassed that of the SOTA methods, with further improvement in the Pittsburgh250k, Pittsburgh30k, and SF-XL-Val test sets focusing on changes in viewpoints, and especially in the Tokyo24/7, Nordland, and SF-XL-Testv1 test sets with changes in complex appearance environments.

### Ablation studies

#### Number of mix layers

**Fig. 5:**
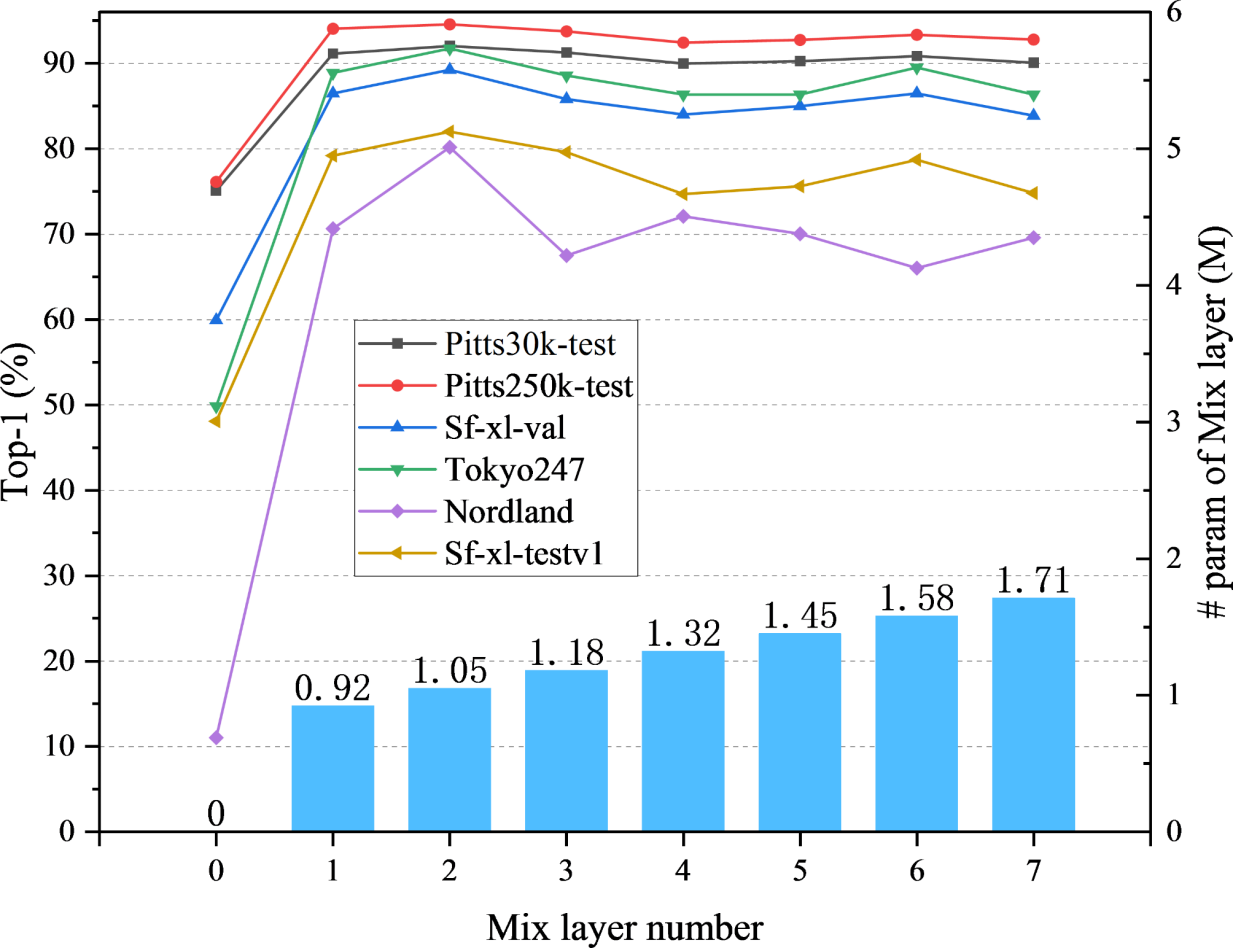
Ablation on the number of feature mix blocks for each dataset.

In DINO-Mix, the number of layers *L* in the feature mixer is also critical for image retrieval accuracy. To determine the optimal number of mixer layers, we conducted tests on the Pitts30k-test, Pitts250k-test, Sf-xl-val, Tokyo24/7, Nordland, and Sf-xl-testv1 datasets with different numbers of mix layers *L* (1–7) for DINO-Mix using ViTb14 as the backbone. The TOP-1 accuracy is depicted in Fig. 5. A careful examination of the figure reveals that DINO-Mix exhibited a lackluster test accuracy without any mix layers across all six datasets. However, upon incorporating one mix layer, test accuracy was substantially improved. This observation highlights the pivotal role played by the feature aggregation module in elevating the precision of DINO-Mix. As the number of mix layers increased to two, there was a marginal improvement in test accuracy, where it peaked. As the number of mix layers continued to increase, DINO-Mix’s test accuracy on the six datasets displayed a slow decline with fluctuations, accompanied by a linear parameter increase. Based on the above analysis, this study adopted a two-layer mix scheme as the optimal feature aggregator in DINO-Mix.

#### Descriptor dimensionality

**Fig. 6:**
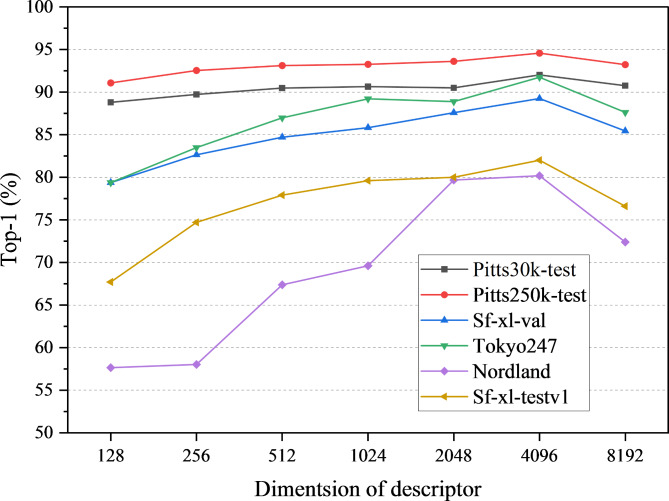
Ablation on the dimensionality levels for each dataset.

We conducted an ablation study on the dimensionality of image feature vectors extracted using the DINO-Mix model. The experiment used ViTb14, which exhibited the best performance as the backbone, with two layers in the mixer module. The test datasets used were the Pitts30k-test, Pitts250k-test, Sf-XL-val, Tokyo24/7, Nordland, and Sf-XL-testv1, and the image feature vector dimensionality was varied by changing the number of channels in the output vector of the mixer module. The tested dimensions of the image feature vectors were 128, 256, 512, 1,024, 2,048, 4,096, and 8,192. As depicted in Fig. 6, an increase in the dimensionality of image feature vectors was observed to positively impact the overall Top-1 test accuracy of DINO-Mix across all the datasets. This trend was particularly pronounced in the SF-XL-val, Tokyo24/7, Nordland, and SF-XL-testv1 datasets, where accuracy rapidly increased. Ultimately, the highest accuracy was achieved at a dimensionality of 4,096. This phenomenon suggests that using image feature vectors with a dimensionality that is too low may result in reduced robustness to variations such as changes in illumination and seasonal shifts in VPR tasks. Consequently, we adopted a final image feature dimensionality of 4,096 in this study.

#### Backbone architecture

**Fig. 7:**
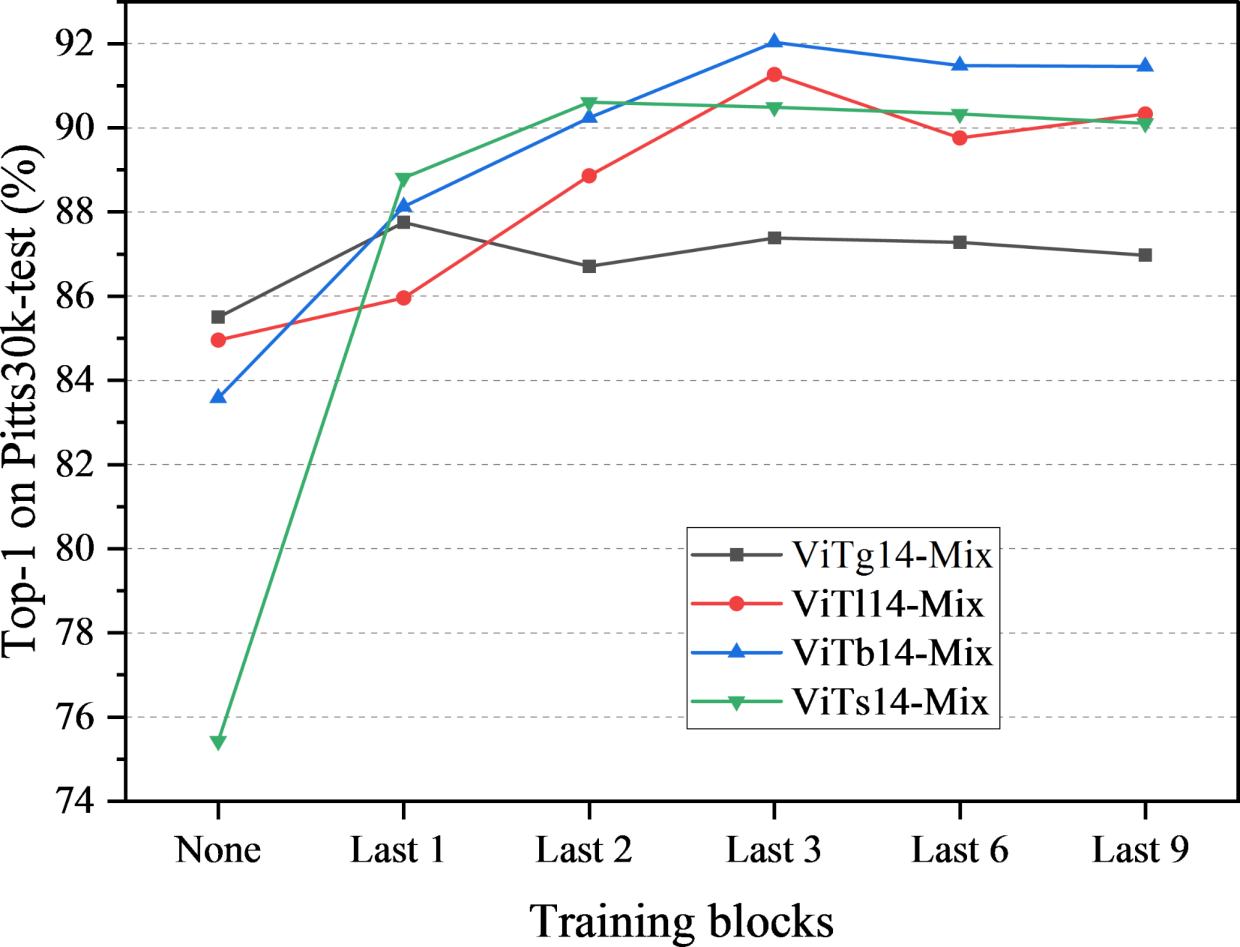
DINO-Mix models with different weights for updating the number of layers.

DINOv2 encompasses four ViT models, with ViTg14 (giant) being the largest. Through model knowledge distillation, three smaller models were obtained from the distillation process, including ViTl14 (large), ViTb14 (base), and ViTs14 (small), as displayed in Table 1. To evaluate the performance of these four models in the DINO-Mix framework, we conducted training with GSV-Cities as the training set and tested the Pitts30k-test dataset using ViTg14-Mix, ViTl14-Mix, ViTb14-Mix, and ViTs14-Mix. The feature mixer was fixed at two layers, and the dimensionality of the image feature vectors was set to 4096. In addition, we trained and tested the DINO-Mix models with four different backbone networks under six scenarios: updating the weights of the last one, two, three, six, and nine blocks and not updating the weights of the backbone (none). The results are shown in Fig. 7.

From the perspective of the four differently sized backbones, ViTb14-Mix exhibited higher accuracy than the other three models, with a maximum Top-1 accuracy of 92.03%. In contrast, ViTg14-Mix exhibited the worst overall performance. This suggests that ViTg14’s large parameter count extracted deeper features from images, adversely affecting subsequent feature aggregation in the feature mixer.

Models without parameter updates for the backbone demonstrated poorer performance. As the number of updated blocks increased, ViTb14-Mix and ViTl14-Mix gradually improved test accuracy, reaching their highest values after updating the parameters of the last three blocks and stabilizing. In contrast, ViTs14-Mix achieved the highest test accuracy and stability after updating the parameters of the last two blocks. However, for ViTg14-Mix, the block parameter updates did not significantly enhance accuracy. Starting from the last three blocks, the ViTg14-Mix test accuracy showed a downward trend. This indicates that excessively deep block parameter updates may alter the original pre-trained parameters extensively.

Additionally, we evaluated the parameter counts (Params), floating-point operations (FLOPs), and average inference times (Avg. inference time) across six test sets for the four DINO-Mix models, as presented in Table 4. Vitg14-Mix and ViTl14-Mix exhibit high parameter counts, FLOPs, and extended average inference times. Conversely, ViTb14-Mix and ViTs14-Mix demonstrate significantly reduced parameter counts and FLOPs, with average inference times of only 10.3 ms and 10.2 ms, respectively. These results indicate that ViTb14-Mix and ViTs14-Mix can effectively meet the speed requirements for most VPR tasks.

In summary, updating the parameters of the last three blocks of the backbone yielded optimal results. We selected ViTb14-Mix, which has a moderate parameter count, low FLOPs, breakneck inference speed, and superior test accuracy, as the final model for DINO-Mix.

**Table 4:** Params, FLOPs, and average inference time for DINO-Mix models.

Model	Params (MB)	FLOPs (T)	Avg. inference time (ms)
ViTg14-Mix	1,136.1	467.5	319.1
ViTl14-Mix	304.2	125.4	113.3
ViTb14-Mix	86.6	35.8	10.3
ViTs14-Mix	22.7	9.2	10.2

### Image retrieval comparison

**Fig. 8:**
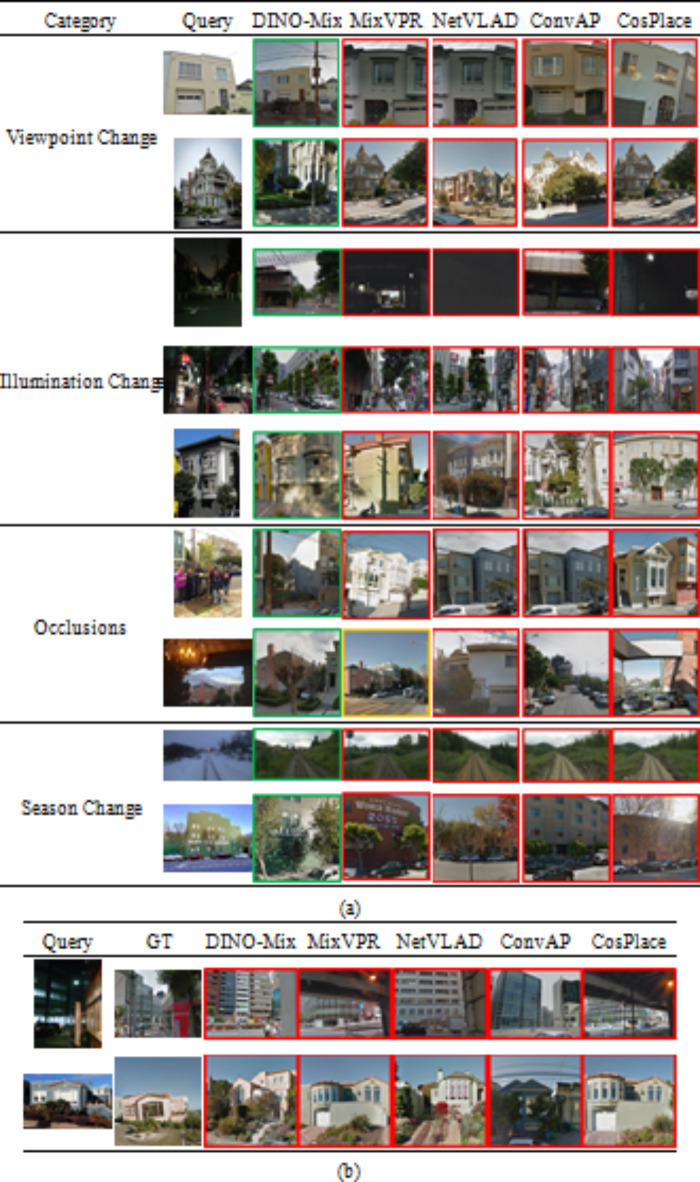
Comparison of VPR results (Top-1) of DINO-Mix with other methods in complex cases. (**a**) Successful VPR cases of DINO-Mix. (**b**) Failed VPR cases of DINO-Mix. The green and red boxes in the table represent image retrieval success and failure, respectively. The yellow box represents correct image content, but the localization distance exceeds the threshold *s*.

In this study, we compared the performance of DINO-Mix with those of SOTA methods, comprising MixVPR, NetVLAD, ConvAP, and CosPlace, in image retrieval tasks. To demonstrate the robustness of DINO-Mix for VPR in complex environments, we selected several representative image retrieval examples from the Tokyo24/7, SF-XL-Testv1, and Nordland datasets. We presented four challenging scenarios: viewpoint changes, illumination changes, object occlusions, and seasonal variations. In these examples, DINO-Mix succeeded, whereas the other methods failed to accurately locate the query image, as shown in Fig. 8.

#### Viewpoint change

Viewpoint changes encompass variations in the field angle and range, posing image retrieval challenges. Rows 1 and 2 in Fig. 8 (a) show viewpoint changes in field angle and field range. Only DINO-Mix resisted the interference caused by viewpoint changes and retrieved the correct image; the other methods retrieved similar but incorrect buildings or scenes.

#### Illumination change

Illumination change significantly affects image retrieval accuracy. Dim lighting conditions can blur image textures, adversely affecting feature extraction and image retrieval accuracy. Rows 3 in Fig. 8 (a) depict the image retrieval cases under dark conditions. Rows 4 and 5 present nighttime scenarios with artificial and natural light variations, respectively. DINO-Mix exhibited strong robustness against illumination changes, whereas the other methods suffered from the effects of lighting variations and failed to retrieve accurate results.

#### Occlusion

Image retrieval focuses primarily on objects, such as buildings, facilities, and natural landscapes. However, pedestrians, vehicles, and other objects can interfere with the semantic information in an image, posing challenges for image retrieval. As shown in rows 6 in Fig. 8 (a), where many pedestrians are present in the query images, and in row 7, where the influence of buildings is significant, these occlusions pose significant difficulties for image retrieval. MixVPR retrieved the correct content but exceeded the threshold (25 m) in the localization results. In contrast, despite these challenges, DINO-Mix successfully extracted the correct features from the images and retrieved accurate results.

#### Season change

The appearance characteristics of locations undergo significant changes in different seasons, such as heavy snowfall in winter (as illustrated in row 8 of Fig. 8 (a)) and leaves falling from trees (row 9). These seasonal variations also have a profound impact on image retrieval accuracy. Under such challenging circumstances, DINO-Mix overcame the drastic contrast caused by seasonal changes and achieved satisfactory results.

#### Failure cases

DINO-Mix cannot perform adequately in specific challenging scenarios. As illustrated in Fig. 8(b), the query image in the initial row was captured in the evening, with a narrow viewing angle and a blurry image—this significantly impaired DINO-Mix’s capacity to extract features, resulting in retrieval failure. The query image and the ground truth (GT) in the second row were taken at different periods, and the color of the same building has changed, which is evident that DINO-Mix cannot exclude the interference of such factors, leading to retrieval and localization failure. Other VPR techniques also falter in retrieving with precision. Therefore, image enhancement for VPR tasks and how to overcome the effects of scene changes over ample periods on VPR are directions worthy of further research.

Figure 8. Comparison of VPR results (Top-1) of DINO-Mix with other methods in complex cases. (a) Successful VPR cases of DINO-Mix. (b) Failed VPR cases of DINO-Mix. The green and red boxes in the table represent image retrieval success and failure, respectively. The yellow box represents correct image content, but the localization distance exceeds the threshold *s*.

### Attention map visualization

**Fig. 9:**
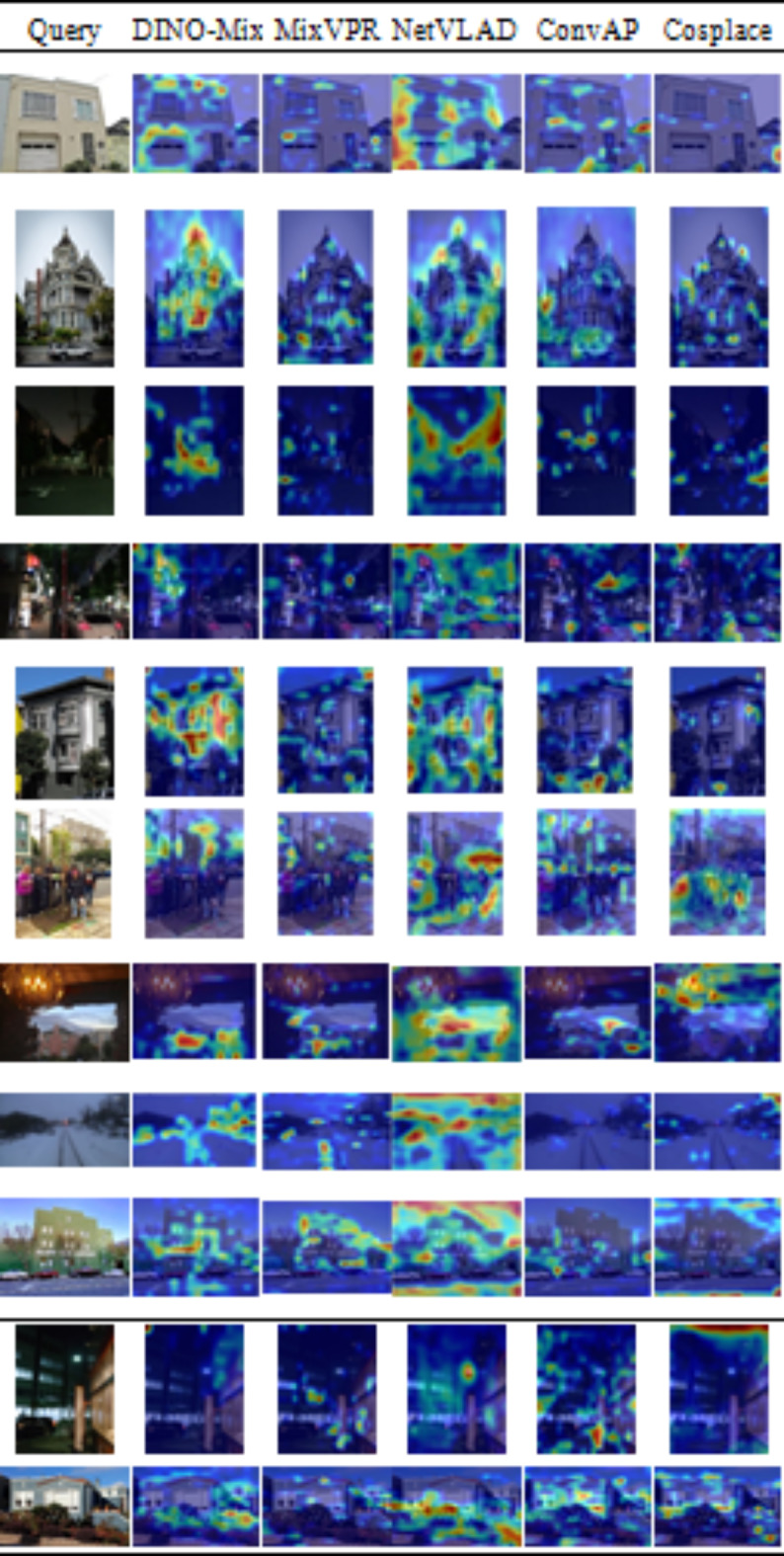
Attention map visualization of the query image.

To provide a more intuitive demonstration of the superiority of DINO-Mix over other VPR methods, we visualized their attention maps as presented in Fig. 9. The attention scores are represented by varying colors from blue to green to red, indicating low to high attention levels. Our analysis reveals that DINO-Mix can focus more on buildings, object contours, and textures, which are crucial for image retrieval. In contrast, it effectively excludes negative elements such as pedestrians, cars, and occlusions. This suggests that DINO-Mix has a greater ability to capture essential features and extract more robust image representations. However, as shown in Fig. 9, row 10, the blurring and inadequate brightness of the image make it difficult for DINO-Mix to notice valuable features, which leads to retrieval failure. This deficiency is the direction in which the model needs to be improved.

## Conclusions

In this study, we proposed a novel VPR framework called DINO-Mix. First, we modified and fine-tuned the structure of the DINOv2 model. We then converted the extracted features from the backbone into feature maps and used the mix feature aggregation module to aggregate these feature maps to obtain global feature vectors. The experimental results on different test sets demonstrate that the proposed DINO-Mix model outperforms SOTA methods regarding VPR accuracy, with an average improvement of 5.14% across test sets containing challenging conditions. Furthermore, through a series of image retrieval examples under difficult circumstances, we demonstrated that the performance of the DINO-Mix architecture significantly surpasses that of current SOTA architectures. However, our experiments revealed that DINO-Mix exhibits poor performance in scenarios involving dynamic blurring and inadequate brightness of images. Furthermore, scene changes occurring over extended periods diminished the accuracy of DINO-Mix. Consequently, our future work will involve delving into the image enhancement techniques for the VPR task and incorporating specialized training to address scene changes over ample periods, aiming to enhance VPR accuracy.

## Data Availability

The datasets generated and/or analysed during the current study are available in the DINO-Mix repository, https://github.com/GaoShuang98/DINO-Mix.
